# Central sensitization predicts greater fatigue independently of musculoskeletal pain

**DOI:** 10.1093/rheumatology/kez028

**Published:** 2019-02-27

**Authors:** Katie L Druce, John McBeth

**Affiliations:** 1 Arthritis Research UK Centre for Epidemiology, Division of Musculoskeletal & Dermatological Sciences, University of Manchester, Manchester, UK; 2 NIHR Manchester Musculoskeletal Biomedical Research Unit, Central Manchester University Hospitals NHS Foundation Trust, Manchester, UK

**Keywords:** fatigue, pain, central sensitization

## Abstract

**Objectives:**

To test whether central sensitization was associated with greater fatigue, independently of musculoskeletal pain.

**Methods:**

2477 prospective cohort study participants completed a baseline questionnaire comprising the Chalder Fatigue Scale (CFQ), pain, demographics, physical activity, anxiety, depression and medication use. In a clinical assessment of 290 (11.7%) participants, central sensitization was measured by the wind-up ratio test at the hand (WUR-H) and foot (WUR-F). Bioelectric impedance determined proportion body fat. All participants were followed up 12 months later, at which time they completed the CFQ. Linear regression, with inverse probability sampling weights, tested the relationship between WUR at baseline and CFQ at 12 months, adjusted for baseline CFQ, demographics, lifestyle factors, mental health and baseline pain.

**Results:**

At baseline, the median interquartile range WUR-H and WUR-F were similar (2.3 (1.5, 4.0) and 2.4 (1.6, 3.9) respectively) and did not differ by sex (difference WUR-H: −0.29, 95% confidence interval −1.28–0.71; WUR-F: −0.57 (−1.50–0.36) or age(WUR-H: −0.53, −1.49–0.43; WUR-F:−0.08, −0.98–0.82). WUR-H scores (β = 0.11, 95% confidence interval: 0.07–0.16) and WUR-F scores (0.13, 0.08–0.17) were positively associated with CFQ scores at follow-up, independently of baseline CFQ and other covariates. These associations were not explained by baseline pain.

**Conclusion:**

Fatigue was predicted by central sensitization, independently of the presence of pain. For those seeking to treat fatigue, the benefit of interventions that reduce central sensitization should be investigated.

**Rheumatology key messages**

- Fatigue is common in inflammatory and non-inflammatory MSK disorders and persists despite disease remission.
- Central sensitization significantly predicts fatigue, independently of MSK pain, demographics, lifestyle factors and mental health.
- Centrally acting medications and non-pharmacological interventions may be beneficial for those seeking improved fatigue management.

## Introduction

Fatigue is the subjective experience of intense tiredness or exhaustion, often unrelated to energy exertion and not relieved by rest [1]. Fatigue is common across people with musculoskeletal **(**MSK) disorders. Up to 80% of people with inflammatory mediated diseases such as rheumatoid arthritis, ankylosing spondylitis and systemic lupus erythematosus [2–4] have fatigue. Inflammation has been proposed as a causal mechanism [5] and treatment with anti-inflammatory drugs reduces fatigue in excess of required minimum clinically important differences in between 65% and 75% of patients [6, 7]. However, fatigue remains common among those with inflammatory disease despite disease remission [3, 8], with as many as 63% of people with rheumatoid arthritis reporting fatigue in excess of general population norms, while in DAS 28 remission [8]. Fatigue is also common in non-inflammatory MSK disorders, such as fibromyalgia, osteoarthritis, neck and lower back pain [2, 9, 10].

Central sensitization is the amplification of sensory input across multiple systems and may be characterized by amplified pain responses, unpleasant sensations to physical stimulus, including heat and mechanical stimuli, and heightened sensitivity to environmental stimuli, including light and sound [11, 12]. Central sensitization is a putative common fatigue mechanism that could explain the high rates of fatigue across MSK disorders [13]. However the relationship is likely to be confounded by MSK pain. Central sensitization is a known MSK pain mechanism [11] and, in turn, MSK pain appears to be mechanistically associated with fatigue [14, 15]. Here we test the hypothesis that, in an unselected general population, sample central sensitization would be associated with fatigue, and that the relationship would be independent of the putative confounding effect of MSK pain (see Supplementary Fig. S1, available at *Rheumatology* online).

## Methods

### Overview

Data were obtained from 2477 participants enrolled in the Pain Across the Adult Life Span (PAALS) study. Data collection methods have been described elsewhere [16], but briefly, PAALS was a prospective population-based cohort study of adults in the north of England. At baseline, participants completed a postal questionnaire that included information about fatigue, pain, demographics, lifestyle factors and mental health (response rate 73% (2477/3379)). At a clinic visit, a random sample of participants underwent testing for central sensitization and all participants were followed up at 12 months to complete a second postal questionnaire. PAALS received full ethical approval from the North West 8 Local Research Ethics Committee (10/H1013/29) and the Research Ethics Committee of the University of Manchester and participants provided informed consent at the time of enrolment to PAALS.

### Baseline questionnaire

*Fatigue*

Fatigue was measured using the 11-item Chalder Fatigue Scale (CFQ) [17]. The 11 scale items were scored on a 4-point scale ranging from ‘less than usual’ to ‘much worse than usual’ and summed to create a total score of fatigue ranging between 0 and 33.

*MSK pain*

Participants recorded whether they had any ache or pain that lasted for one day or longer in the past month. Those who reported pain were asked to shade any areas of a blank body manikin in which they had experienced the pains. The pain information reported was coded using the Manchester Scoring Template, to create a score 0 (no pain) to 29 (pain in all sites of the body).

*Demographics and lifestyle factors*

Participants reported their sex and date of birth, from which age was calculated. Physical activity (PA) was captured by the Rapid Assessment of PA [18], scored from 0 (no PA) to 7 (20 min or more a day of vigorous physical activities, three or more days a week) and such that scores <6 indicate a suboptimal level of PA. Participants were also asked to list any current medications they were on using a free-text box. Reported medications were coded and total number of distinct medications was recorded for each participant.

*Mental health*

Anxiety and depression were measured by the Hospital Anxiety and Depression Scale [19]. The 14 Hospital Anxiety and Depression Scale items were scored on a 4-point scale, summed to create a score ranging from 0–21 for each sub-scale, where higher scores indicate a greater burden of symptoms [19].

### Clinical assessment

A total of 290 (11.7%) attended a clinical assessment. To assess central sensitization, participants had a wind-up ratio test at the thenar eminence of the right hand (WUR-H) and dorsum of the left foot (WUR-F). The WUR test determines the perceived intensity of pain reported of a single pin prick (using 256 mN punctate probe), compared with the perceived intensity of pain reported after series of 10 consecutive pin pricks conducted at 1 s intervals within an area of 1 cm^2^. Pain intensity was rated using a 0–100 numerical rating scale, where 0 indicated no pain and 100 indicated the most intense pain imaginable. The WUR is calculated as the perceived intensity of a series of pinpricks/the intensity of a single pin prick. The assessments were conducted by two members of the PaALS research team (a research physiotherapist and a research fellow in quantitative sensory testing), who were trained by quantitative sensory testing experts to follow a standardized protocol [20]. The Spearman’s rho for between-day variation in WUR was 0.64, indicating acceptable reliability. Further, additional reliability checks were performed by an independent observer who was present for at least three assessments per assessor and ensured they performed the assessment in line with the standard protocol. Bioelectric impedance (Tanita BC-418 Segmental Body Composition Analyzer, Tanita Europe B.V., Amsterdam, The Netherlands) determined proportion body fat.

### Follow-up questionnaire

All baseline participants were asked to complete a follow-up questionnaire 12 months after they completed the baseline questionnaire. The questionnaire included the CFQ.

### Analysis

Participant characteristics were examined and the characteristics of those who participated in the clinical assessment were compared with all participants. The relationship between central sensitization (WUR-H: Model 1; WUR-F: Model 2) and fatigue was tested in three stages. First, univariable linear regression, with inverse probability sampling weights, tested the relationship between baseline central sensitization and fatigue at 12 months, adjusted for age and sex. Inverse probability sampling weights were calculated such that those who undertook clinical assessments represented all participants in the cohort study. Sample weights were proportional to the inverse of the probability of an observation being sampled and are an indicator of how many of the participants in the cohort were represented by each participant for whom a clinical assessment was conducted. In stage 2, the univariable model was further adjusted for MSK pain and CFQ score at baseline. Finally, a multivariable model tested the relationship adjusted for all putative confounders. Results are expressed as beta coefficients, which denote the change in fatigue score associated with a one-unit increase in the predictor, and 95% confidence intervals (95% CI). All analyses were conducted in STATA 14.0.

## Results

### Participants

The baseline characteristics of all participants, and only those who completed the clinical assessment are shown in Table 1. In brief, participants tended to be female (63%) with a median age of 64 (interquartile range 55–74 years). The median fatigue (12, 11–16), pain (3, 0–8), depression (4, 2–7) and anxiety (5, 2–8), scores indicated a moderate level of symptom burden. There were no significant differences between those who returned a completed questionnaire and those who participated in the clinical study. The median (interquartile range) WUR-H and WUR-F were similar (2.3 (1.5, 4.0) and 2.4 (1.6, 3.9) respectively), and did not differ by sex (difference WUR-H: −0.29, 95% CI −1.28–0.71; WUR-F: −0.57 (−1.50–0.36) or age (WUR-H: -0.53, -1.49–0.43; WUR-F:-0.08, −0.98–0.82).

**Table 1:** Baseline characteristics of participants in the PAALS study

	All participants (*n* = 2477)	Participants who completed clinical assessment and provided full data (*n* = 194)
Female, *n* (%, 95% CI)	1549, (63, 61, 65)	122, (63, 56, 70)
Age, median (IQR)	64 (55–74)	66.5 (59–78)
Fatigue (CFQ, 0–33), median (IQR) (*n*=2272)	12 (11–16)	12 (11–16)
MSK Pain (number of sites 0–29), median (IQR) (*n*=2463)	3 (0–8)	3 (0–6)
Depression (HADS; 0–21), median (IQR) (*n*=2285)	4 (2–7)	4 (2–7)
Anxiety (HADS; 0–21), median (IQR) (*n*=2271)	5 (2–8)	5 (2–7)
Sub-optimal PA, *n* (%, 95% CI) (*n*=2346)	1278 (55, 53, 57)	112, (58, 51, 65)
Number of current medications, median (IQR)	2 (0–5)	3 (1–5)
WUR-H, median (IQR)	—	2.3 (1.5, 4.0)
WUR-F, median (IQR)	—	2.4 (1.6, 3.9)
% Body fat, median (IQR)	—	31.8 (25.1–38.5)

95% CI: 95% confidence interval; CFQ: Chalder Fatigue Scale; HADS: Hospital Anxiety and Depression Scale; RAPA: Rapid Assessment of Physical Activity; IQR: interquartile range; MSK: musculoskeletal; WUR-H: wind-up ratio test at the hand; WUR-F: wind-up ratio test at the foot; PAALS: Pain Across the Adult Life Span; PA: Physical Activity.

A total of 2122 participants returned the follow-up questionnaire. Non-responders were younger (median age: 60, interquartile range: 50–78 *vs* 65, 56–74) and a greater proportion were categorized in the most deprived (31.0% (95% CI: 26.4–36.1), *vs* 22.1% (20.3–23.9)), compared with responders. There was no difference between the groups with respect to the proportion of women (non-responders 63.7% (58.5–68.5), responders 62.4% (60.3–64.4)), nor baseline fatigue scores (median score: 13, 11–17 *vs* 12, 11–16).

### Predictors of fatigue

After adjusting for age and sex, WUR-H (β = 0.17, 95% CI 0.10–0.2395%) and WUR-F (0.18, 0.12–0.24) was associated with CFQ at follow-up. The relationship was independent, but attenuated by the inclusion of baseline MSK pain and CFQ (Model 1—WUR-H: 0.10, 0.05–0.24; Model 2—WUR-F: 0.12, 0.07–0.17). In a fully adjusted model, WUR-H (0.11, 0.07–0.16) and WUR-F (0.13, 0.08–0.17) predicted CFQ at follow-up, independently of baseline MSK pain (0.02, −0.01–0.05; 0.01, −0.03–0.04, respectively), CFQ (0.43, 0.38–0.47; 0.43, 0.38–0.47, respectively) and all other putative confounders (baseline fatigue, depression, anxiety, PA, body fat and number of medications). Anxiety and PA at baseline were independent associated with CFQ at follow-up (Table 2).

**Table 2:** Baseline predictors of fatigue at 12 months

	Model 1 – Hand (β, 95% CI)			Model 2 – Foot (β, *P*-value)		
	Stage 1 – ‘univariable’ model^a^	Stage 2 – partially adjusted model^a^	Stage 3 – fully adjusted model^a^	Stage 1 – ‘univariable’ model^a^	Stage 2 – partially adjusted model^a^	Stage 3 – fully adjusted model^a^
WUR-H	**0.17 0.10, 0.23**	**0.10, 0.05, 0.24**	**0.11, 0.07, 0.16**	**0.18, 0.12–0.24**	**0.12, 0.07–0.17**	**0.13, 0.08–0.17**
MSK pain (number of sites 0–29)		**0.04, 0.01, 0.07**	0.02, -0.01, 0.05		0.03, 0.001–0.07	0.01, -0.03–0.04
Baseline fatigue (CFQ, 0–33)		**0.52, 0.49, 0.56**	**0.43, 0.38, 0.47**		**0.52, 0.48–0.55**	**0.43, 0.38–0.47**
Depression (HADS; 0–21)			−0.04, −0.10, 0.02			-0.04, -0.10–0.26
Anxiety (HADS; 0–21)			**0.20, 0.14, 0.25**			**0.20, 0.14–0.26**
PA (RAPA; 0–7)			**-0.27, -0.35, -0.19**			**-0.23, -0.32– -0.15**
% body fat			0.01, -0.02, 0.03			0.01, -0.01–0.03
N of current medications			0.02, -0.4, 0.07			0.03, -0.03–0.09

a age and sex adjusted; bold denotes statistical significance. 95% CI: 95% confidence interval; CFQ: Chalder Fatigue Scale; HADS: Hospital Anxiety and Depression Scale; RAPA: Rapid Assessment of Physical Activity; MSK: musculoskeletal; WUR-H: wind-up ratio test at the hand; PA: Physical Activity.

## Discussion

This study sought to determine the relationship between central sensitization and CFQ. We have demonstrated that central sensitization, as measured by WUR-H or WUR-F, significantly predicts fatigue, independently of MSK pain, demographics, lifestyle factors and mental health.

There are several limitations that should be considered when interpreting these results. First, due to available resources, only a sub-sample of participants attended the clinical assessment and had WUR and proportion body fat data available. Nevertheless, the use of sample weighting allowed these data to be applied to the whole cohort. Due to the multidimensional nature of fatigue, it is possible that a number of key-covariates were not collected in this study; for example, we note that our analysis included only the number, not type, of medications being used by participants and were therefore not able to specifically adjust for the use of mediations known to be associated with fatigue (e.g. steroids, antidepressants). It is therefore likely that residual confounding exists. That being said, a key strength of this study is that a number of including pain, anxiety and depression [2–4], were adjusted for. Furthermore, by conducting this analysis within a relatively large population, and adjusting for baseline MSK pain, this study has overcome some important methodological issues levied against previous studies [12].

Though previous studies exist in this area, the results are conflicting, and most have sought to investigate the role of central sensitization in pain among fatigued populations, rather than to delineate its specific role in fatigue [12, 13, 21]. We propose that by overcoming these issues, our results contribute to an improved understanding of why fatigue occurs in such a breadth of conditions, and why reported fatigue may not necessarily correlate with levels of inflammation or respond to immunosuppression [3, 8, 9]. Fatigue was predicted by central sensitization, independently of the presence of pain. In light of the fact that there are no licensed or recommended therapies for fatigue, these findings suggest that medications and non-pharmacological interventions may be beneficial for improved fatigue management. Such interventions may include those that are commonly used in chronic pain, including PA (e.g. aquatic exercise and yoga), behavioural interventions and pharmacological approaches such as antidepressants (e.g. amitriptyline) [22, 23].

## Supplementary Material

kez028_Supplementary_Figure
