# Supplementary figures and images for: Central sensitization predicts greater fatigue independently of musculoskeletal pain
Source: Rheumatology (Oxford). 2019 Feb 27;58(11):1923–7. doi: 10.1093/rheumatology/kez028 (PMC6812719; doi:10.1093/rheumatology/kez028)

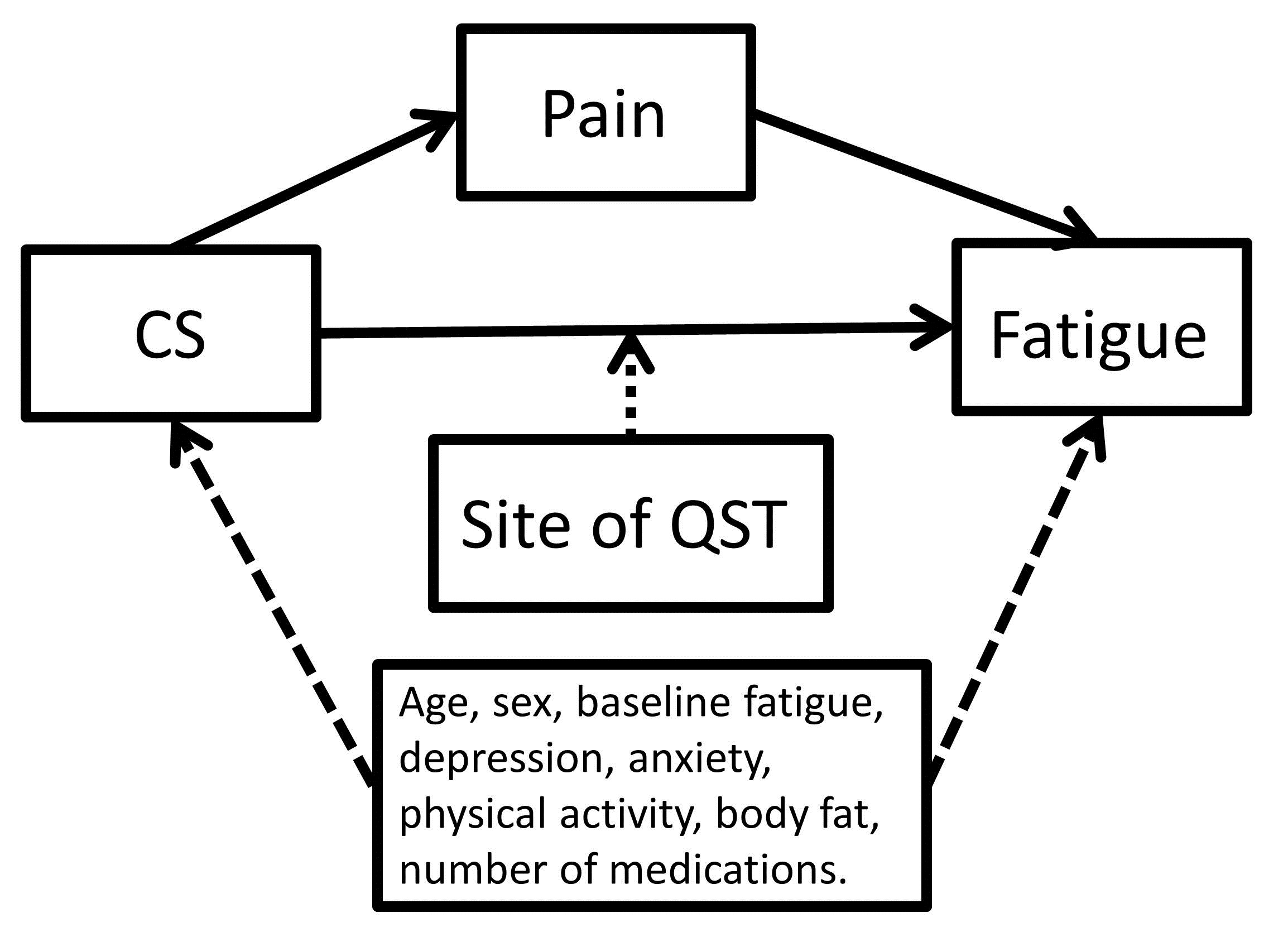

Supplement: kez028_Supplementary_Figure [file kez028_supplementary_figure.jpeg]
